# *Hemicycliophora ahvasiensis* n. sp. (Nematoda: Hemicycliophoridae), and data on a known species, from Iran

**DOI:** 10.21307/jofnem-2020-128

**Published:** 2021-02-02

**Authors:** Sedighe Azimi, Joaquín Abolafia, Majid Pedram

**Affiliations:** 1 Department of Plant Protection, Faculty of Agriculture, Shahid Chamran University of Ahvaz, Ahvaz, Iran; 2 Departamento de Biología Animal, Biología Vegetal y Ecología, Universidad de Jaén, Campus de las Lagunillas, Avenida de Ben Saprut s/n. 23071-Jaén, Spain; 3 Department of Plant Pathology, Faculty of Agriculture, Tarbiat Modares University, Tehran, Iran

**Keywords:** D2-D3-LSU, Hemicycliophora, H. conida, ITS, morphology, morphometrics, phylogeny, sheath nematode, taxonomy

## Abstract

*Hemicycliophora ahvasiensis* n. sp., recovered from the rhizospheric soil of date palm in Khuzestan province, southwest Iran, is described and illustrated based upon morphological, morphometric and molecular data. The new species is characterized by its sheath, closely fitting most of the body, cuticle with or without numerous irregular lines, sometimes appearing as blocks in distal body region. Lateral field without discrete longitudinal lines, but often with continuous broken striae or anastomoses. Continuous lip region with single annulus, slightly elevated labial disc, stylet with posteriorly sloping knobs. Vulva with or without slightly modified lips, spermatheca with sperm and tail conoid, symmetrically narrowing at distal region to form a narrow conical region. Morphologically, the new species looks similar to *H. indica*, *H. labiata*, *H. siddiqii*, *H. tenuistriata* and *H. typica*. The latter species appears more similar to the new species under light microscopy, but could be separated using the scanning electron microscopy and molecular data. The new species was also compared with *H. epicharoides* and *H. dulli*, two species with close phylogenetic affinities to it. The phylogenetic relationships of the new species were reconstructed and discussed using partial sequences of the D2-D3 expansion segments of large subunit, and internal transcribed spacer regions (LSU D2-D3 and ITS rDNA). *Hemicycliophora conida*, the second studied species, was recovered from north Iran and characterized by morphological and molecular data.

In their excellent contribution to the systematics of the superfamily Hemicycliophoroidea Skarbilovich, 1959 (Siddiqi, 1980), Chitambar and Subbotin (2014) reviewed the taxonomy of the genus *Hemicycliophora* (De Man, 1921) and updated data of the currently valid species. In the same year, Subbotin et al. (2014) addressed aspects of the pathogenicity of *Hemicycliophora* species on associated host plants, the difficulties of morphological identifications due to morphological plasticity, and the lack of scanning electron microscopic (SEM) and molecular data. Currently the genus contains 133 species (132 listed in Chitambar and Subbotin, 2014 and one in Maria et al., 2018).

There are 12 species of *Hemicycliophora* have been reported from different provinces in Iran. They are *H. belemnis* Germani & Luc, 1973, *H. chilensis* Brzeski, 1974, *H. conida* Thorne, 1955, *H. iranica* Loof, 1984, *H. lutosa* Loof & Heyns, 1969, *H. megalodiscus* Loof, 1984, *H. poranga* Monteiro & Lordello, 1978, *H. ripa* Van den Berg, 1981, *H. sculpturata* Loof, 1984, *H. spinituberculata* Loof, 1984, *H. sturhani* Loof, 1984 and *H. vaccinii* Reed & Jenkins, 1963. All of these species were characterized using traditional taxonomic methods (Eskandari, 2018). In an effort to document *Hemicycliophora* species occurring in Iran, two populations were recovered from soil samples obtained from different geographical locations in northern and southern regions. The preliminary morphological studies revealed the population recovered from south Iran resembled *H. typica* de Man, 1921 under light microscope (LM), but further studies using SEM and molecular data, and comparisons with all known species of the genus, revealed it to be an unknown species, described herein as *H. ahvasiensis* n. sp. The second species recovered from north Iran belonged to *H. conida* Thorne, 1955.

## Materials and methods

### Nematode extraction and morphological observations

Several soil samples were collected from date palm and fruit tree gardens in Khuzestan and Gilan provinces, Iran. The relevant information of the presently studied nematode populations, and those included in phylogenetic analyses, are given in Table 1. Jenkins’ method (Jenkins, 1964) was used to extract the nematodes from soil samples. The collected specimens were killed in hot 4% formaldehyde solution and transferred to anhydrous glycerin according to De Grisse (1969). Observations and measurements were conducted using a Leitz SM-LUX light microscope equipped with a drawing tube. Some of the specimens were photographed using an Olympus DP72 digital camera attached to an Olympus BX51 light microscope equipped with differential interference contrast (DIC).

**Table 1.** Information of the species/populations of *Hemicycliophora* studied in present paper and those of ingroup and outgroup taxa used in phylogenetic analyses.

			GenBank accession numbers		
Species	Host	Locality	D2-D3 of LSU rDNA	ITS	Reference or identifier
*H. ahvasiensis* n. sp.	*Phoenix dactylifera*	Khuzestan province, Iran	MT901580, MT901581	MT901582, MT901583	Present study
*H. californica*	*Salix* sp.	Yolo County, CA, USA	KF430518, KF430519	KF430576	Subbotin et al. (2014)
*H. conida*	*Punica granatum*	Gilan province, Iran	–	MT901584	Present study
*H. conida*	Unknown plant	Belgium	FN433875	–	I. Tandingan De Ley et al. (unpub.)
*H. conida*	Turf grasses	Football pitch, Madrid, Spain	KF430447	KF430580	P. Castillo; Subbotin et al. (2014)
*H. conida*	Unknown plant	Clallam County, WA, USA	KF430448	KF430579	Subbotin et al. (2014)
*H. dulli*	Peat	South Africa	MT329669, MT329670	MT329671, MT329672	M. Rashidifard (unpub.)
*H. epicharoides*	*Ammophila arenaria*	Serranova, Brindisi, Italy	KF430512	–	Subbotin et al. (2014)
*H. epicharoides*	*Ammophila arenaria*	S. Barrameda, Cádiz, Spain	–	KF430608	Subbotin et al. (2014)
*H. epicharoides*	*Pragmites* sp.	Epiros, Greece	–	KF430606	Subbotin et al. (2014)
*H. floridensis*	*Pinus elliotti*	Lake City, FL, USA	KF430506	KF430536	Subbotin et al. (2014)
*H. gracilis*	*Prunus domestica*	Hamilton City, Glenn County, CA, USA	KF430480	KF430562	Subbotin et al. (2014)
*H. gracilis*	*Prunus domestica*	Butte City, Glenn County, CA, USA	KF430481	–	Subbotin et al. (2014)
*H. gracilis*	Unknown plant	Brooklyn Park, MN, USA	KF430482	–	Subbotin et al. (2014)
*H. gracilis*	Unknown plant	California, USA	–	FN435301	I. Tandingan De Ley et al. (unpub.)
*H. gracilis*	Unknown plant	Sacramento County, CA, USA	–	MG019827	Van den Berg et al. (2018)
*H. halophila*	*Desmoschoenus spiralis*	Taylors Mistake, New Zealand	KF430444, KF430445	KF430582, KF430583	Subbotin et al. (2014)
*H. hellenica*	*Arundo donax*	Filippias, Epirus, Greece	KF430453	KF430584	Subbotin et al. (2014)
*H. iberica*	*Populus nigra*	Arroyo Frío, Jaén, Spain	KF430461	KF430539, KF430540	Subbotin et al. (2014)
*H. iberica*	*Quercus suber*	Hinojos, Huelva, Spain	KF430462	–	Subbotin et al. (2014)
*H. iberica*	*Quercus suber*	Santa Elena, Jaén, Spain	KF430463	KF430541	Subbotin et al. (2014)
*H. italiae*	*Ammophila arenaria*	Zapponeta, Foggia, Italy	KF430458	–	Subbotin et al. (2014)
*H. labiata*	*Poa pratensis*	South Korea	MK305971, MK305972	MK305973, MK305974	Mwamula et al. (2020)
*H. lutosa*	Unknown plant	Gauteng province, South Africa	GQ406240, GQ406241	GQ406237	Van den Berg et al. (2010)
*H. lutosoides*	Turf grasses	Madrid, Spain	KF430454	–	Subbotin et al. (2014)
*H. lutosoides*	*Juncus* sp.	Cádiz, Spain	–	KF430537, KF430538	Subbotin et al. (2014)
*H. obtusa*	*Pinus pinea*	Moguer, Huelva, Spain	KF430521	KF430578	Subbotin et al. (2014)
*H. onubensis*1	*Pinus pinea*	Moguer, Huelva, Spain	KF430449, KF430450	KF430587, KF430588	Subbotin et al. (2014); Van den Berg et al. (2018)
*H. parvana*2	Turf grasses	New Hanover County, NC, USA	KF430501	–	Subbotin et al. (2014); Van den Berg et al. (2018)
*H. parvana*2	Turf grasses	Carteret County, NC, USA	KF430502	–	Subbotin et al. (2014); Van den Berg et al. (2018)
*H. parvana*2	Bentgrass	Texas, USA	KC329574	KC329575	Ma and Agudelo (2015)
*H. parvana*	*Prunus persica*	Punta Gorda, FL, USA	MG019825	–	Van den Berg et al. (2018)
*H. parvana*2	*Andropogon virginicus*	Paines Praire, FL, USA	–	KF430524, KF430526	Subbotin et al. (2014); Van den Berg et al. (2018)
*H. parvana*2	Turf grasses	New Hanover County, NC, USA	–	KF430528	Subbotin et al. (2014); Van den Berg et al. (2018)
*H. poranga*	*Poa annua*	Monterey County, CA, USA	KF430432, KF430434	KF430598	Subbotin et al. (2014)
*H. poranga*	Turf grasses	San Francisco, CA, USA	MG019815	–	Van den Berg et al. (2018)
*H. poranga*	Unknown plants	Marin County, CA, USA	MG019816	–	Van den Berg et al. (2018)
*H. poranga*	*Salix* sp.	Santa Rosa, CA, USA	–	KF430590	Subbotin et al. (2014)
*H. poranga*	*Apium graveolens*	Argentina	–	KF430596	Subbotin et al. (2014)
*H. poranga*	*Lepidorrhachis mooreana*	San Francisco, CA, USA	–	KF430600	Subbotin et al. (2014)
*H. raskii*	Grasses	Sacramento County, CA, USA	KF430520	KF430577	Subbotin et al. (2014)
*H. robbinsi*3	Turf grasses	Brunswick, NC, USA	KF430488, KF430492	–	Subbotin et al. (2014); Van den Berg et al. (2018)
*H. robbinsi*3	Turf grasses	Indian Hills, CA, USA	KF430491	–	Subbotin et al. (2014); Van den Berg et al. (2018)
*H. robbinsi*3	Turf grasses	San Antonio, TX, USA	–	KF430544	Subbotin et al. (2014); Van den Berg et al. (2018)
*H. robbinsi*3	*Borrichia* sp.	St Augustine, FL, USA	–	KF430550	Subbotin et al. (2014); Van den Berg et al. (2018)
*H. robbinsi*3	*Phoenix roebelenii*	Fort Lauderdale, FL, USA	–	KF430552	Subbotin et al. (2014); Van den Berg et al. (2018)
*H. signata*	Grasses	Chemba District, Mozambique	MG019824	–	Van den Berg et al. (2018)
*H. similis*	*Fragaria x ananassa*	Cartaya, Huelva, Spain	KF430465	–	Subbotin et al. (2014)
*H. subbotini*	*Cinnamomum camphora*	Zhejiang Province, China	MG701275–MG701277	MG701272, MG701273	Maria et al. (2018)
*H. thienemanni*	*Salix* sp.	Moscow, Russia	KF430469–KF430471	KF430570–KF430572	Subbotin et al. (2014)
*H. thienemanni*	*Populus nigra*	Castillo de Locubin, Jaén, Spain	–	KF430568	Subbotin et al. (2014)
*H. thornei*	*Vitis vinifera*	La Rambla, Córdoba, Spain	KF430452	KF430581	Subbotin et al. (2014)
*H. typica*	Grasses	Gauteng province, South Africa	KF430515	KF430603	Subbotin et al. (2014)
*H. typica*	Sugarcane	South Africa	–	GQ406238, GQ406239	Van den Berg et al. (2010)
*H. vaccinii*	*Pinus pinaster*	Carnota, Coruña, Spain	–	KF430542	Subbotin et al. (2014)
*H. vaccinii*	*Pinus pinaster*	Monteagudo Isl., Pontevedra, Spain	KF430459, KF430460	–	Subbotin et al. (2014)
*H. vidua*	*Camellia* sp.	South Carolina, USA	–	JQ708147	Cordero López et al. (2013)
*Hemicycliophora* sp.	Unknown plant	Iran	KY284835	–	E. Miraeiz, R. Heydari (unpub.)
*Hemicycliophora* sp. 1	Grasses	Terovo, Epirus, Greece	AY780974	KF430602	Subbotin et al. (2005); Subbotin et al. (2014)
*Hemicycliophora* sp. 2	Unknown plant	Birdlings Flat, New Zealand	KF430516, KF430517	KF430609, KF430610	Subbotin et al. (2014)
*Hemicycliophora* sp. 3	*Zea mays*	Tingle Farms, Willcox, AZ, USA	–	KF430573, KF430574	Subbotin et al. (2014)
*Hemicycliophora* sp. 5	Turf grasses	Carteret County, NC, USA	–	KF430575	Subbotin et al. (2014)
*Hemicycliophora* sp. 6	*Nothofagus* forest	Kaitoke Waterworks, New Zealand	KF430446	KF430585, KF430586	Subbotin et al. (2014)
*Hemicycliophora* sp. 7	*Pinus pinea*	Almonte, Huelva, Spain	KF430451	KF430589	Subbotin et al. (2014)
*Hemicycliophora* sp. 8	Unknown plant	Henrieville, UT, USA	KF444173	–	Subbotin et al. (2014)
*Hemicycliophora* sp. 8	Turf grasses	Monterey, CA, USA	KF430494	KF430559	Subbotin et al. (2014)
*Hemicycliophora* sp. 9	*Trifolium repens*	Preveza, Greece	KF430509, KF430511, KF430514	KF430605	Subbotin et al. (2014)
*Hemicycliophora* sp. 9	*Agrostis* sp.	Jaroslavl region, Russia	–	KF430604	Subbotin et al. (2014)
*Hemicycliophora* sp. 9	Unknown plant	Brake, Germany	AY780973	–	Subbotin et al. (2005); Subbotin et al. (2014)
*Hemicycliophora* sp. 10	*Salix* sp.	Yolo County, CA, USA	KF430483, KF430485–KF430486	KF430566, MG019828	Subbotin et al. (2014); Van den Berg et al. (2018)
*Hemicycliophora* sp. 11	*Andropogon virginicus*	Paines Prairie, FL, USA	KF430493	KF430557, KF430558	Subbotin et al. (2014)
*Hemicycliophora* sp. 12	Grasses	Saint Paul, MN, USA	KF430474	–	Subbotin et al. (2014)
*Hemicycliophora* sp. 12	Unknown plant	Brooklyn Park, MN, USA	KF430475	–	Subbotin et al. (2014)
*Hemicycliophora* sp. 12	Unknown plant	Sedona, AZ, USA	KF430476	–	Subbotin et al. (2014)
*Hemicycliophora* sp. 13	*Neoregelia* sp.	Los Angeles County, CA, USA	KF430507, KF430508	–	Subbotin et al. (2014)
*Hemicycliophora* sp. 15	Unknown plant	Vicinity of Trois–Rivières, Quebec, Canada	MG019819	–	Van den Berg et al. (2018)
*Hemicycliophora* sp. 16	Unknown tree	east of Temecula, CA, USA	MG019818	MG019829	Van den Berg et al. (2018)
*Hemicycliophora* sp. 17	Unknown tree	Pismo Beach, San Luis Obispo County, CA, USA	–	MG019830	Van den Berg et al. (2018)
*Hemicycliophora* sp. 18	Unknown plant	Vicinity of Quebec City, Quebec, Canada	MG019820	–	Van den Berg et al. (2018)
*Gracilacus bilineata*	*Bambusa* sp.	Taiwan	–	EU247525	Chen et al. (2008)
*Paratylenchus bukowinensis*	Unknown plant	Monopoli, Italy	AY780943	–	Subbotin et al. (2005)
*Paratylenchus minutus*	*Annona squamosa*	Taiwan	–	EF126180	Chen et al. (2009)
*Paratylenchus nanus*	Unknown plant	Niebüll, Germany	AY780946	–	Subbotin et al. (2005)
*Trophotylenchulus floridensis*	*Pinus elliottii*	Crystal river, Florida, USA	–	JN112261	Tanha Maafi et al. (2012)

Note: ^1^Originally identified as *H. ripa* ^2^Originally identified as *H. wyei* ^3^Originally identified as *Hemicycliophora* sp. 4.

### Scanning electron microscopy (SEM)

Specimens preserved in glycerin were selected for observation according to Abolafia (2015). They were hydrated in distilled water, dehydrated in a graded mixture of ethanol-acetone series, critical point-dried with liquid carbon dioxide, and coated with gold. The mounts were examined with a Zeiss Merlin microscope (5 kV).

### DNA extraction, PCR and sequencing

For molecular analyses, single female specimens were picked out, examined in a drop of distilled water on a temporary slide under the light microscope, transferred to 3 μl of TE buffer (10 mM Tris-Cl, 0.5 mM EDTA; pH 9.0) on a clean slide, and then crushed using a cover slip. The suspension was collected by adding 20 μl TE buffer. One DNA sample for the Gilan population and two DNA samples for the Khuzestan population were prepared in this manner. The DNA samples were stored at –20°C until used as a PCR template. Primers for LSU rDNA D2-D3 ampliﬁcation were forward primer D2A (5’–ACAAGTACCGTGAGGGAAAGT–3’) and reverse primer D3B (5’–TCGGAAGGAACCAGCTACTA–3’) (Nunn, 1992). Primers for ampliﬁcation of ITS rDNA were forward primer TW81 (5’–GTTTCCGTAGGTGAACCTGC–3’) and reverse primer AB28 (5’–ATATGCTTAAGTTCAGCGGGT–3’) as described in Vovlas et al. (2008). The 25 μl PCR mixture contained 14.5 μl of distilled water, 3 μl of 10 × PCR buffer, 0.5 μl of 10 mM dNTP mixture, 1.5 μl of 50 mM MgCl2, 1 μl of each primer (10 pmol/μl), 0.5 μl of *Taq* DNA polymerase (Cinna Gen, Tehran, Iran, 5 U/μl), and 3 μl of DNA template. The thermal cycling program was as follows: denaturation at 95°C for 4 min, followed by 35 cycles of denaturation at 94°C for 30 s, annealing at 52°C for 40 s, and extension at 72°C for 80 s. A ﬁnal extension was performed at 72°C for 10 min. Amplification success was evaluated by electrophoresis on 1% agarose gel (Aliramaji et al., 2018, 2020). The PCR products were purified using the QIAquick PCR purification kit (Qiagen®) following the manufacturer’s protocol and sequenced directly using the PCR primers with an ABI 3730XL sequencer (Bioneer Corporation, South Korea). The newly obtained sequences of the studied species were deposited into the GenBank database (accession numbers LSU D2-D3 MT901580/MT901581 and ITS rDNA MT901582 /MT901583 for the new species and MT901584 for ITS rDNA of *H. conida*, as indicated in Table 1).

**Table 2.** Morphometrics of *Hemicycliophora ahvasiensis* n. sp. from Khuzestan province, Iran.

Character	Female holotype	Female paratypes	Juvenile
n	1	20	1
L	868.7	830.3 ± 48.3 (767–893)	600
a	22.2	21.5 ± 2.1 (17.9–24.5)	18.0
b	5.9	5.8 ± 0.3 (5.4–6.5)	4.8
c	10.7	10.1 ± 1.2 (8.3–11.5)	9.8
c'	2.9	3.0 ± 0.3 (2.5–3.5)	2.6
o	11.5	12.2 ± 0.9 (9.4–15.3)	9.0
DGO	7.9	8.1 ± 0.9 (7.4–10.0)	5.5
V	85.5	84.1 ± 0.9 (82.6–85.5)	–
St	68.4	66.5 ± 2.3 (63.3–71.0)	60.8
m	81.5	80.6 ± 1.4 (77.4–83.7)	80.7
Stylet knob height	4	4.1 ± 0.5 (4–5)	3.8
Stylet knob width	7	6.6 ± 0.7 (6–8)	6.6
Excretory pore from anterior end	171	168.3 ± 6.1 (159–180)	168
Diam. at mid-body	39	38.4 ± 3.6 (32–46)	33
Diam. at anus (ABD)	27	26.8 ± 1.7 (24–29)	23
Diam. at vulva	38	38.4 ± 2.1 (35–43)	–
Vulva-anterior body distance	744	700 ± 43 (653–751)	–
Vulva-tail terminus distance	125	129.5 ± 6.0 (113–142)	–
Spermatheca-vulva distance	89	87.6 ± 13.2 (74–121)	–
Lip diam.	15	15.7 ± 0.9 (14–18)	14
Lip height	7	6.7 ± 0.7 (6–9)	6
First body annulus diam.	16	16.9 ± 0.9 (15–19)	15
Second body annulus diam.	18	18.6 ± 1.1 (16–21)	16
Pharynx length	145	142.6 ± 4.8 (134–151)	125
Annulus width	4	4.1 ± 0.3 (3.4–4.7)	2.8
Tail length	81	83.3 ± 7.9 (74–92)	61
V-anus distance	45	47.9 ± 9.9 (32–64)	–
R	245	221.3 ± 8.6 (212–247)	216
RSt	19	19.5 ± 1.1 (18–21)	22
Rph	41	41.1 ± 3.2 (35–48)	46
Rex	48	47.4 ± 3.6 (42–50)	58
RV(ant)	193	185.3 ± 8.4 (167–198)	–
RV	52	47.8 ± 6.9 (38–59)	–
RVan	15	15.0 ± 3.8 (10–22)	–
Ran	37	32.9 ± 4.9 (25–47)	–
VL/VB	3.3	3.4 ± 0.3 (2.8–3.9)	–
Spermatheca length	29	19.8 ± 5.4 (14–29)	–
Spermatheca diam.	15	15.5 ± 1.6 (12–22)	–
St%L	7.8	7.9 ± 0.4 (7.5–8.4)	10

### Phylogenetic analyses

The newly obtained sequences of the D2-D3 fragments of LSU rDNA of the both populations, and the selected sequences from GenBank, were aligned by Clustal X2 (http://www.clustal.org/) using the default parameters. The ITS dataset was aligned using MUSCLE as implemented in MEGA6 (Tamura et al., 2013). The editing of both alignments was performed manually. The outgroup taxa were chosen according to previous studies (Subbotin et al., 2014; Van den Berg et al., 2018; Maria et al., 2018). The base substitution model was selected using MrModeltest 2 (Nylander, 2004) based on the Akaike information criteria. A general time reversible model, including among-site rate heterogeneity and estimates of invariant sites (GTR + G + I), was selected for the both phylogenies.

The Bayesian analysis was performed to infer the phylogenetic trees using MrBayes v3.1.2 (Ronquist and Huelsenbeck, 2003), running the chains for two million generations. After discarding burn-in samples and evaluating convergence, the remaining samples were retained for further analyses. The Markov chain Monte Carlo (MCMC) method within the Bayesian framework were used to determine equilibrium distribution and help estimate the posterior probabilities of the phylogenetic trees (Larget and Simon, 1999) using the 50% majority rule. Bayesian posterior probability (BPP) values higher than 0.50 are given on appropriate clades. The output ﬁles of the phylogenetic program was visualized using Dendroscope v3.2.8 (Huson and Scornavacca, 2012) and re-drawn in CorelDRAW software version 17.

## Results

### Systematics

**Figure 1:**
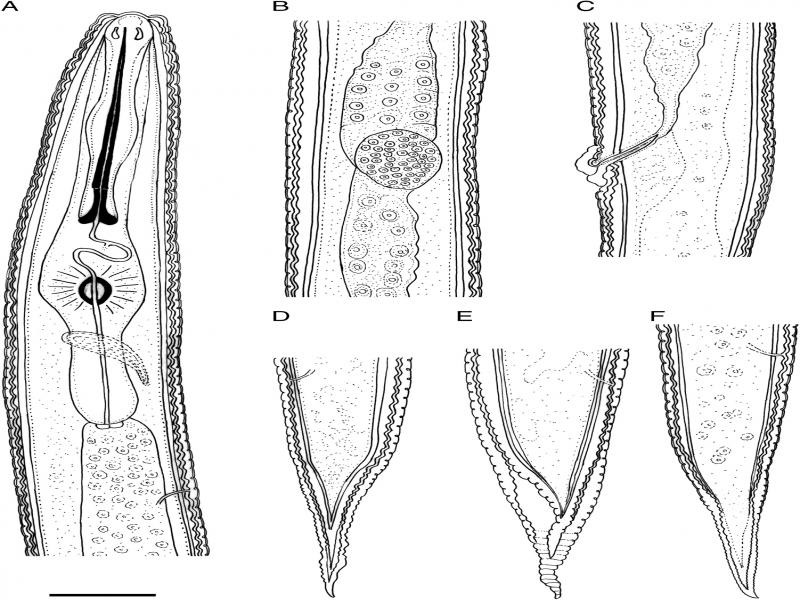
Line drawings of *Hemicycliophora ahvasiensis* n. sp. Female. A: Anterior body region; B: Spermatheca; C: Vulval region; D–F: Variation of posterior body end morphology. (Scale bar = 20 *μ*m).

**Figure 2:**
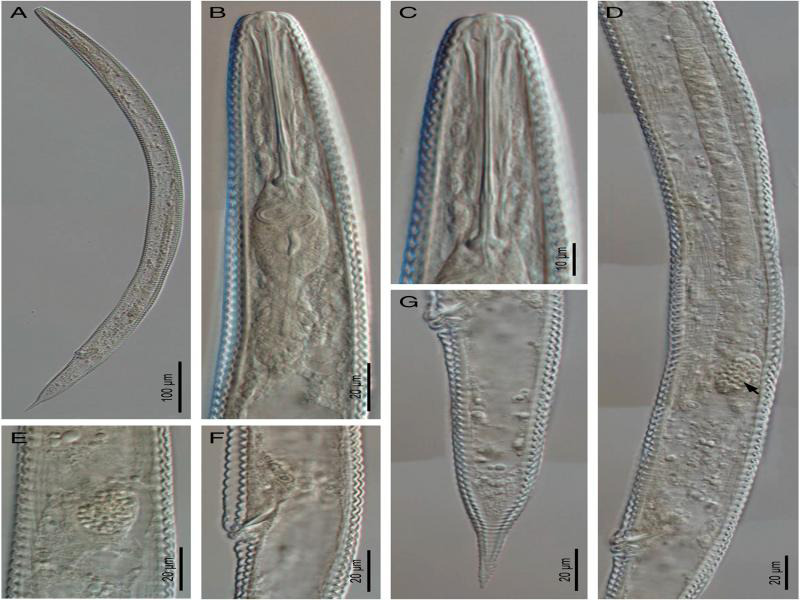
Light photomicrographs of *Hemicycliophora ahvasiensis* n. sp. Female. A: Entire body; B, C: Anterior body region; D: Reproductive system (the arrow indicates the spermatheca); E: Spermatheca; F: Vagina; G: Posterior body region.

**Figure 3:**
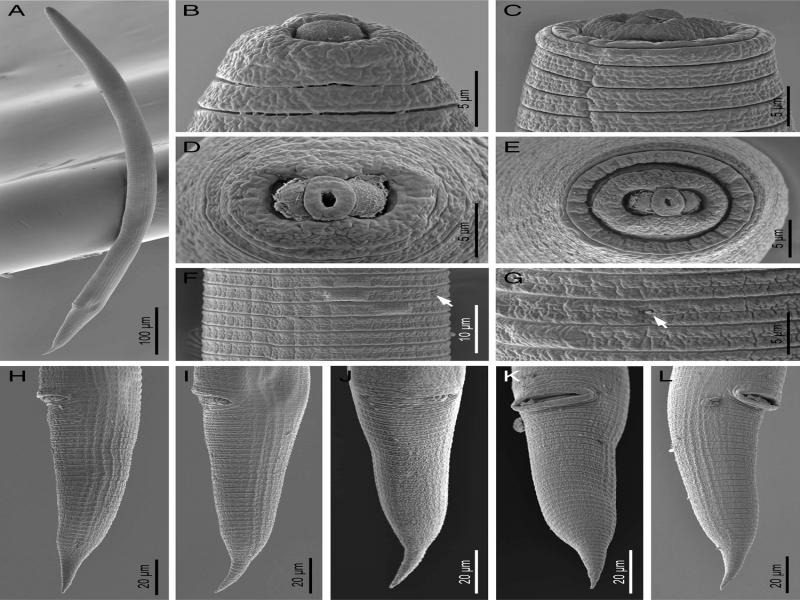
Scanning electron micrographs of *Hemicycliophora ahvasiensis* n. sp. Female. A: Entire body; B, C: Anterior end showing labial region; D, E: *En face* view of labial area; F, G: Annuli ornamentation (the arrows indicate the excretory pore); H–L: Posterior body region.

**Figure 4:**
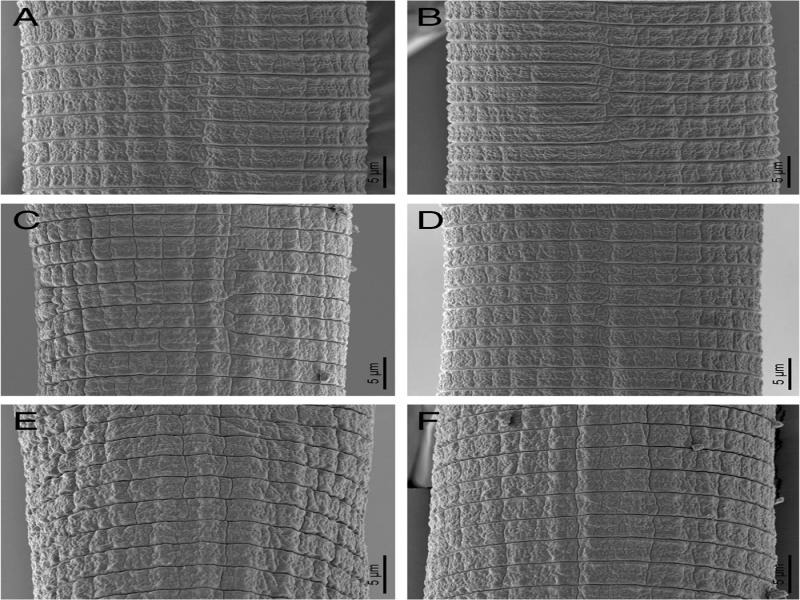
Scanning electron micrographs of *Hemicycliophora ahvasiensis* n. sp. Female. A–F: Mid-body annuli ornamentation.

*Hemicycliophora ahvasiensis n. sp.*(Figures 1–4; Table 2).

### Description

#### Female

Body straight to slightly ventrally arcuate following heat fixation. Cuticular sheath closely appressed over entire or most of body. Under LM, annuli rounded, with or without longitudinal lines, appearing as blocks mostly in the distal body region. Block-like differentiations are more prominent in distal body region under SEM. Lateral field with no longitudinal lines, but having broken or continuous striae or anastomoses. Amphidial openings large, partly plugged. Lip region continuous with body contour, bearing one wide annulus. Labial disc slightly elevated. Stylet with posteriorly sloping knobs, having moderate to large cavity at base. Pharynx criconematoid, with pharyngeal corpus absent, metacorpus (median bulb) ovoid bearing central valves, short isthmus surrounded by the nerve ring and reduced pyriform basal bulb. Cardia short, surrounded by intestinal tissue. Excretory pore five to 10 annuli posterior to the pharynx base. Hemizonid indistinct. Reproductive system monodelphic-prodelphic, outstretched, composed by long ovary with oocytes arranged in one or two rows, spermatheca round to oval, ﬁlled with spheroid sperm cells, vulva with not or slightly modified lips, vulval sleeve slightly elongate, one to two annuli long. Body portion behind vulva slightly narrowing towards distal region. Distance between vulva to anus about five anal body diam. Tail conoid, symmetrically narrowing at about 35% of its length at distal region to form a narrower conical section ending to a finely rounded to sharp terminus.

#### Male

Not found.

#### Juvenile

One juvenile specimen was found in the population that is similar to female except by a smaller body size and undeveloped sexual organs.

#### Type host and locality

This population was recovered from the rhizospheric soil of date palm (*Phoenix dactylifera* L.) collected from Ahvaz city in Khuzestan province, southwest Iran. The GPS information of the sampling site is 31°18´11.1˝N, 48°39´10.1˝E.

#### Etymology

The specific epithet of the new species refers to the original city name in Latin where it was discovered.

#### Type material

The holotype and 12 paratype females were deposited into the nematology laboratory of the Department of Plant Protection, Shahid Chamran University of Ahvaz, Ahvaz, Iran. Three paratype females deposited at the Wageningen Nematode Collection (WaNeCo), Wageningen, The Netherlands. Two paratype females deposited at the Nematode Collection of the Department of Animal Biology, Plant Biology and Ecology of the University of Jaén, Jaén, Spain. The ZooBank Life Science Identifier (LSID) for this publication is as follows: http://zoobank.org/urn:lsid:zoobank.org:pub:EEF9C9E9-90B8-4EC1-8BD9-A403FD8D58E4.

#### Diagnosis and relationships

*Hemicycliophora ahvasiensis* n. sp. is mainly characterized by a cuticle with or without longitudinal lines on annuli. Instead of lateral lines there may be broken or continuous striae or anastomoses on lateral sides of the body. The lip region is continuous with body contour and has a single annulus, slightly elevated labial disc, and plugged amphidial openings. Other characters include posteriorly sloping stylet knobs, vulva with or without slightly modified lips and short vulval sleeve, spermatheca full of sperm and conoid tail, symmetrically narrowing at about 35% of its length at the distal region to form a narrower conical region. The polytomous identification codes of the new species from Chitambar and Subbotin (2014) are: A4, B2, C3, D1, E1, F1, G23, H1, I12, J1, K23, L3, M2, N1, O1, P1, Q2, R2, S3, T1, U2, V1, W1, X1, Y-.

In general morphology, the new species is close to *H. indica* Siddiqi, 1961, *H. labiata* Colbran, 1960, *H. siddiqii* Deswal & Bajaj, 1987, *H. tenuistriata* Doucet, 1982 and *H. typica*. A comparison of the new species with the aforementioned species is as follows:

From *H. indica*, by a shorter body (767–893 *vs* 800–1500 μm), lower R, Rph, Rex and RV (212–247 *vs* 270–320, 35–48 *vs* 47–69, 42–50 *vs* 51–67 and 38–59 *vs* 64–81), respectively, lateral field without line(s) (*vs* with three lines), lip region with one annulus (*vs* two or three annuli), short vulval sleeve (*vs* elongate) and tail symmetrically narrowing at about 35% of its length at distal region to form a narrower conical section (*vs* uniformly narrowing).

From *H. labiata*, by annuli with or without longitudinal lines (*vs* not), lateral field lacking line(s) (*vs* having one line), lip region with one annulus (*vs* two or three annuli), short vulval sleeve (*vs* moderately long) and body not constricted immediately posterior to vulva (*vs* constricted).

From *H. siddiqii*, by lateral field lacking line(s) (*vs* having one line), a longer body (767–893 *vs* 650–780 μm), lower a ratio (17.9–24.5 *vs* 27–31), higher c ratio (8.3–11.5 *vs* 7), longer stylet (63.3–71.0 *vs* 57–59 μm), posteriorly located excretory pore (159–180 *vs* 127–146 μm from anterior end), higher R, Rph and Rex (212–247 *vs* 185–198, 35–48 *vs* 30–32 and 42–50 *vs* 35–39, respectively), shorter vulval sleeve (*vs* moderately elongate) and tail symmetrically narrowing at about 35% of its length at distal region to form a narrower conical section (*vs* uniformly narrowing).

From *H. tenuistriata*, by shorter stylet (63.3–71.0 *vs* 70–79 μm), posteriorly located excretory pore (159–180 *vs* 136–158 μm from anterior end), higher R, Rph, RV(ant) and Rex (212–247 *vs* 179–205, 35–48 *vs* 31–37, 167–198 *vs* 146–162 and 42–50 *vs* 33–40, respectively), shorter vulval sleeve (*vs* moderately elongate) and vulval lips not modified (*vs* modified, well developed, extending posteriorly).

From *H. typica*, by cuticle lacking distinct blocks (*vs* having blocks), lateral field lacking line(s) (*vs* having two lines), body not constricted immediately posterior to vulva (*vs* constricted) and short vulval sleeve (*vs* moderately elongate).

From *H. epicharoides* Loof, 1968, a species with close phylogenetic affinities in both LSU and ITS phylogenies, by higher R, RV(ant) and Rex (212–247 *vs* 144–209, 167–198 *vs* 129–167 and 42–50 *vs* 32–43, respectively), lip region with one annulus (*vs* two or three annuli), lower St%L (7.5–8.4 *vs* 9–11), excretory pore located posterior to pharynx base (*vs* anterior or posterior) and tail symmetrically narrowing at about 35% of its length at distal region to form a narrower conical section (*vs* cylindroid anteriorly, mostly narrowing to a bluntly triangular or wedge-shaped posterior part).

From *H. dulli* Van den Berg & Tiedt, 2001, a species with close phylogenetic affinities in ITS phylogeny, by shorter stylet (63.3–71.0 *vs* 73–79 μm), lower Rst (18–21 *vs* 21–25), lateral field lacking line(s) (*vs* having one or two lines), lip region continuous with one annulus (*vs* set off with two annuli), excretory pore located posterior to pharynx base (*vs* anterior or posterior) and vulva with not or slightly modified lips (*vs* vulval lips elongated).

### Hemicycliophora conida Thorne, 1955(

Figure 5; Table 3).

**Figure 5:**
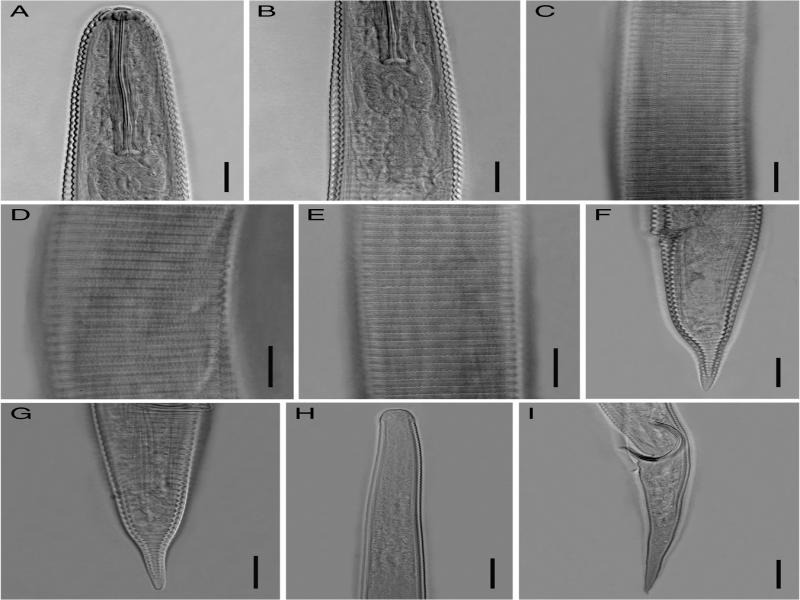
Light photomicrographs of *Hemicycliophora conida* Thorne, 1955 from Gilan province, Iran. A–G: Female. A: Anterior body region; B: Pharyngeal region; C: Lateral field at mid-body; D, E: Annuli ornamentation; F, G: Posterior body region; H, I: Male. H: Anterior body region; I: Posterior body region. (Scale bar = 20 μm).

**Table 3.** Morphometrics of *Hemicycliophora conida* Thorne, 1955 from Gilan province, Iran, and comparison with other population from East Azarbaijan province, Iran.

Reference	Present study		Loof (1984)
Province	Gilan province		East Azarbaijan province
Character	Female	Male	Female
n	10	5	11
L	912.0 ± 21.4 (881–928)	809.0 ± 13.6 (795–822)	820–1020
a	21.2 ± 2.5 (18.6–24.3)	37.3 ± 5.7 (31.8–43.3)	26–30
b	5.3 ± 0.2 (5.2–5.6)	–	5.0–5.6
c	11.6 ± 2.0 (10.1–14.5)	8.2 ± 0.4 (7.8–8.6)	9.7–13.6
c'	2.3 ± 0.4 (1.7–2.6)	5.2 ± 0.5 (4.6–5.6)	–
V	86.7 ± 1.0 (85.5–87.6)	–	86–89
St	92.5 ± 2.1 (90–97)	–	90–103
m	78.3 ± 4.0 (75.3–84.2)	–	–
Stylet knob height	5.0 ± 0.4 (4.3–5.6)	–	–
Stylet knob width	7.8 ± 0.5 (6.9–8.6)	–	–
Excretory pore from anterior end	178.0 ± 8.9 (169–192)	140.2 ± 13.4 (127–164)	–
Diam. at mid-body	43.5 ± 5.5 (38–50)	22 ± 3 (19–25)	–
Diam. at anus/cloaca	35.3 ± 4.3 (30–40)	19 ± 1 (18–20)	–
Diam. at vulva	46.4 ± 5.8 (40–55)	–	–
Vulva-anterior body distance	791 ± 16 (770–809)	–	–
Vulva-tail terminus distance	124.5 ± 7.8 (115–136)	–	–
Spermatheca-vulva distance	82.2 ± 10.1 (72–96)	–	–
Lip diam.	21.3 ± 2.2 (19–24)	10.7 ± 1.5 (9–12)	–
Lip height	7.5 ± 0.6 (7–8)	6.2 ± 0.8 (6–7)	–
First body annulus diam.	23.8 ± 1.9 (20–26)	–	–
Second body annulus diam.	26.1 ± 2.7 (21–30)	–	–
Pharynx length	171.0 ± 4.7 (167–176)	–	–
Annulus width	4.1 ± 0.5 (3.6–5.1)	1.9 ± 0.1 (1.8–2.0)	–
Tail length	88.0 ± 6.5 (79–94)	98.7 ± 6.1 (92–104)	–
V-anus distance	42.0 ± 19.7 (28–71)	–	–
R	230.0 ± 9.2 (224–237)	–	259–286
RSt	21.0 ± 0.9 (18–23)	–	–
Rph	38.0 ± 0.2 (38–39)	–	–
Rex	41.4 ± 1.4 (39–43)	–	48–52
RV(ant)	187.0 ± 3.5 (185–190)	–	207–226
RV	46.0 ± 4.9 (37–54)	–	–
RVan	16.0 ± 8.5 (10–22)	–	11–17
Ran	27.0 ± 2.8 (25–29)	–	35–41
VL/VB	2.7 ± 0.3 (2.2–3.4)	–	4.0–5.3
Spermatheca length	22.7 ± 5.9 (12–30)	–	–
Spermatheca diam.	31.8 ± 8.1 (15–39)	–	–
Spicules length	–	54.3 ± 2.1 (52–56)	–
Gubernaculum length	–	20.3 ± 0.6 (20–21)	–
Bursa length	–	41.7 ± 6.4 (37–49)	–

### Description

#### Female

Body straight or ventrally arcuate. Cuticular sheath fitting closely to loosely to body. Lateral fields with two distinct longitudinal lines forming a band, with irregularities and breaks of striae or with anastomosis, an additional central line appears due to four ellipsoid markings on each annulus forming four blocks. Annuli outside lateral field with scratches. Labial region broad, anterior margins rounded, with two distinct annuli and elevated labial disc. Stylet long and slender, knobs posteriorly directed. Pharynx typical of the genus. Nerve ring encircling isthmus. Excretory pore, four annuli posterior and opposite to pharynx base. Reproductive system monodelphic-prodelphic, outstretched, spermatheca rounded to ovate, ﬁlled with spheroid sperm cells, vulval lips modiﬁed, vulval sleeve absent. Tail conical, symmetrically narrowing at distal region, tip rounded.

#### Male

Cuticle annulation fine at midbody. Lateral fields marked by three longitudinal lines. Labial region distinctly trapezoid. Stylet and pharynx degenerated. Spicules semi-circular, tip slightly recurved. Gubernaculum linear, slightly thickened proximally. Bursa with crenate margin. Tail elongate, uniformly narrowing, annuli at distal end irregular.

#### Host and locality

This population was recovered from the rhizospheric soil of pomegranate (*Punica granatum* L.) collected from Rasht city, Gilan province, in north Iran. The geographical position of the sampling site is N36°54´1.687˝, E49°28´37.923˝.

#### Remarks

*H. conida* was originally described by Thorne (1955) from a sugar beet field in Ireland. It was later reported from several countries (Chitambar and Subbotin, 2014). In the report of Loof (1984), the species was recovered from East Azarbaijan province of Iran. Males, however, were not recovered in this study. Later, the species was again recovered from Azarbaijan province, but no morphometric or morphological data were provided (Barooti, 1998). The presently recovered population agreed well with other populations of the species that have been reported from different regions, based upon the morphometric data and morphology (Chitambar and Subbotin, 2014). The spicules length in The Netherlands populations was measured as 18–29 μm by Loof (1968) (Chitambar and Subbotin, 2014), but it was calculated about 55 μm after the drawings, which is in accordance with the presently studied population.

#### Molecular characterization and phylogenetic relationships

**Figure 6:**
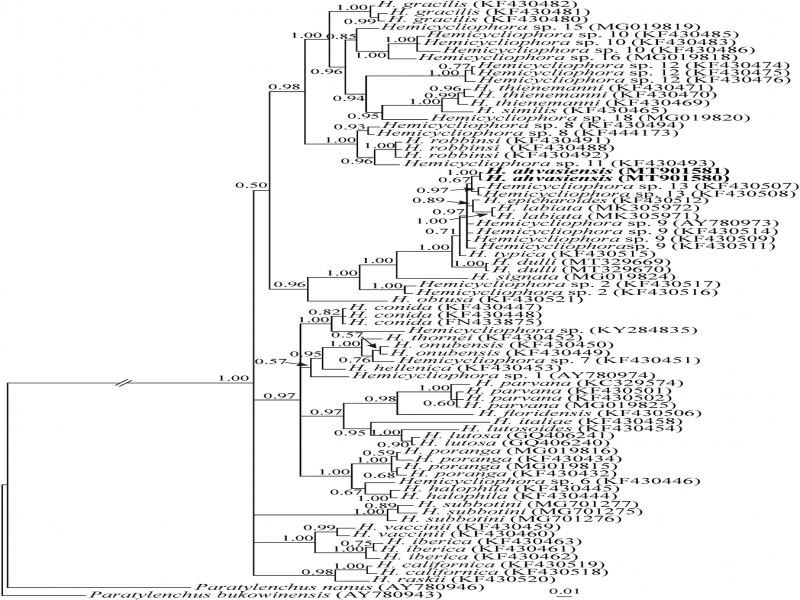
Bayesian 50% majority rule consensus tree inferred from analysis of the D2-D3 domains of the LSU rDNA sequences of *Hemicycliophora ahvasiensis* n. sp. under the GTR + G + I model. (lnL = 6023.6660; freqA = 0.2165; freqC = 0.2342; freqG = 0.3064; freqT = 0.2429; R(a) = 0.4542; R(b) = 1.5000; R(c) = 1.0798; R(d) = 0.4155; R(e) = 4.2300; R(f) = 1; Pinvar = 0.3122; Shape = 0.7157). Bayesian posterior probability values more than 0.50 are given for appropriate clades. New sequences are indicated in bold.

Two 673 and 682 nt long D2-D3 expansion segments of LSU (MT901580, MT901581), one from each female specimen, were generated for the new species. A BLAST search using these sequences revealed they have 99.34% identity with *Hemicycliophora* sp. 9 and *Hemicycliophora* sp. 13 (KF430509 and KF430508, respectively). The efforts to get the LSU sequences of *H. conida* failed. A total of 77 sequences of *Hemicycliophora* spp. and two sequences of *Paratylenchus nanus* Cobb, 1923 and *P. bukowinensis* Micoletzky, 1922 (AY780946 and AY780943, respectively), as outgroup taxa, were selected for a LSU phylogeny. This dataset comprised 750 total characters. The phylogenetic tree inferred using this dataset is presented in Figure 6. The major clade including the new species, also includes *Hemicycliophora* sp. 13 (KF430507, KF430508), the putative closest relative of it, based upon currently available data, *H. epicharoides* (KF430512), *H. labiata* (MK305971, MK305972) and *Helicycliophora* sp. 9 (KF430509, KF430511, KF430514, AY780973). *H. typica* (KF430515) is in a sister relation to the aforementioned major clade.

Two 904 and 907 nt long sequences of ITS rDNA (MT901582, MT901583) were generated for the new species. A single 683 nt long ITS rDNA sequence (MT901584) was obtained for the Iranian population of *H. conida*. A BLAST search using the ITS sequences of the new species revealed they have 98.93% identity with *Hemicycliophora* sp. 9 (KF430605). The BLAST search using ITS sequence of Iranian population of *H. conida* revealed it has 99.55% and 98.21% identity with two other ITS sequences of *H. conida* (KF430580 and KF430579, respectively).

**Figure 7:**
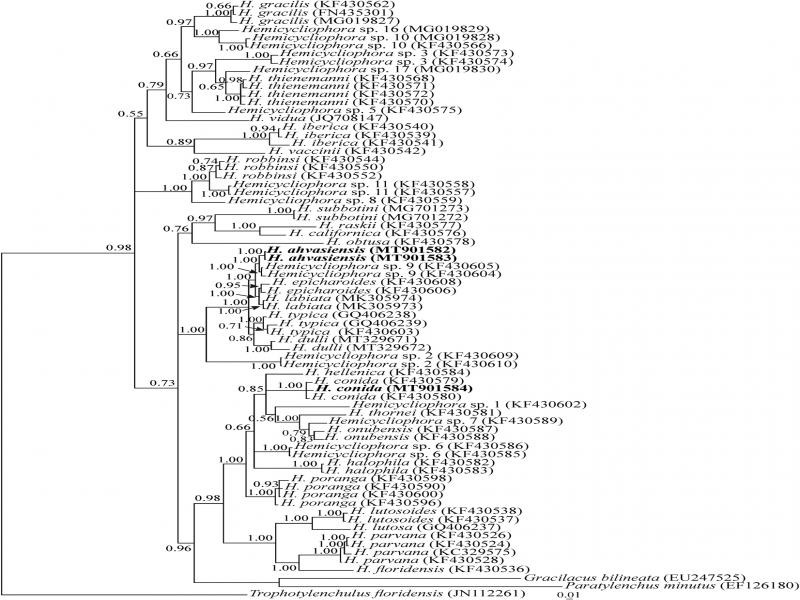
Bayesian 50% majority rule consensus tree inferred from analysis of the ITS rRNA gene of *Hemicycliophora ahvasiensis* n. sp. and Iranian population of *H. conida* under the GTR + G + I model. (lnL = 13525.0293; freqA = 0.2348; freqC = 0.2548; freqG = 0.2520; freqT = 0.2583; R(a) = 1.6876; R(b) = 2.3417; R(c) = 1.8416; R(d) = 0.8470; R(e) = 3.6304; R(f) = 1; Pinvar = 0.1024; Shape = 0.4967). Bayesian posterior probability values more than 0.50 are given for appropriate clades. New sequences are indicated in bold.

A total of 70 sequences of *Hemicycliophora* spp. and three sequences of *Paratylenchus minutus* Linford in Linford, Oliveira & Ishii, 1949, *Trophotylenchulus floridensis* Raski, 1957 and *Gracilacus bilineata* Brzeski, 1995 as outgroup taxa (EF126180, JN112261 and EU247525, respectively) were selected for an ITS phylogeny. This dataset comprised 1164 total characters. The phylogenetic tree inferred using this dataset is presented in Figure 7. The major clade including the new species, also includes *Hemicycliophora* sp. 9 (KF430604, KF430605) that represents the putative closest relative of the new species, *H. epicharoides* (KF430606, KF430608) and *H. labiata* (MK305973, MK305974). The clade including two species *H. typica* (GQ406238, GQ406239, KF430603) and *H. dulli* (MT329671, MT329672) is in sister relation to the aforementioned clade. The ITS sequence of the Iranian isolate of *H. conida* formed a clade with two previously available sequences (KF430579, KF430580) of the species.

## Discussion

The objectives of this study were to characterize one new and one known species of the genus *Hemicycliophora* from Iran. As common in reliable identifications of *Hemicycliophora* spp., the new species was studied using an integrative approach exploiting both morphological (including SEM) and molecular data (Subbotin et al., 2014).

In both inferred LSU and ITS phylogenies, *Hemicycliophora ahvasiensis* n. sp. belonged to a clade including *Hemicycliophora* sp. 9, *H. labiata*, *H. epicharoides*, *H. typica* and *H. dulli*. The close affinity of the aforementioned species was already observed (Subbotin et al., 2014; Van den Berg et al., 2018; Maria et al., 2018; Mwamula et al., 2020).

The newly described species in present study appeared similar to *H. typica* under LM, however, the SEM and molecular data revealed they differ. Sequences of LSU D2-D3, and ITS rDNA sequences of *H. ahvasiensis* n. sp. differed from those of *H. typica* by 6 bp (1.4%) and 31 bp (1.5%), respectively. In the inferred phylogenies, it formed a subclade separate from *H. typica* and other *Hemicycliophora* species.

The new species was isolated from the rhizosphere of date palm tree, that is a major food source for local populations in the Middle East, and plays important roles in their culture and economy (Chao and Krueger, 2007). Additional study is required to clarify if the parasitism of high nematode populations of *H. ahvasiensis* n. sp. can cause damages to this plant.
